# The secret life of ground squirrels: accelerometry reveals sex-dependent plasticity in above-ground activity

**DOI:** 10.1098/rsos.160404

**Published:** 2016-09-28

**Authors:** Cory T. Williams, Kathryn Wilsterman, Victor Zhang, Jeanette Moore, Brian M. Barnes, C. Loren Buck

**Affiliations:** 1 Center for Bioengineering Innovation and Department of Biological Sciences, Northern Arizona University, Flagstaff, AZ 86011, USA; 2 Department of Integrative Biology, University of California Berkeley, Berkeley, CA 94720, USA; 3 Institute of Arctic Biology, University of Alaska Fairbanks, Fairbanks, AK 99775, USA

**Keywords:** accelerometer, activity logger, daily energy expenditure, arctic ground squirrel, *Urocitellus parryii*

## Abstract

The sexes differ in how and when they allocate energy towards reproduction, but how this influences phenotypic plasticity in daily activity patterns is unclear. Here, we use collar-mounted light loggers and triaxial accelerometers to examine factors that affect time spent above ground and overall dynamic body acceleration (ODBA), an index of activity-specific energy expenditure, across the active season of free-living, semi-fossorial arctic ground squirrels (*Urocitellus parryii*). We found high day-to-day variability in time spent above ground and ODBA with most of the variance explained by environmental conditions known to affect thermal exchange. In both years, females spent more time below ground compared with males during parturition and early lactation; however, this difference was fourfold larger in the second year, possibly, because females were in better body condition. Daily ODBA positively correlated with time spent above ground in both sexes, but females were more active per unit time above ground. Consequently, daily ODBA did not differ between the sexes when females were early in lactation, even though females were above ground three to six fewer hours each day. Further, on top of having the additional burden of milk production, ODBA data indicate females also had fragmented rest patterns and were more active during late lactation. Our results indicate that sex differences in reproductive requirements can have a substantial influence on activity patterns, but the size of this effect may be dependent on capital resources accrued during gestation.

## 1. Introduction

In most animals, males and females differ profoundly in how and when they allocate time and energy towards reproduction, and this is expected to influence daily patterns of movement and energy expenditure across the annual cycle. In female mammals, for example, mothers are required to meet both maternal and offspring energy requirements throughout gestation and lactation with daily energy expenditure (DEE) typically peaking during late lactation [1,2]. Females can respond to the energetic demands of reproduction by increasing the absolute amount of time spent foraging, often at the expense of risk-aversive behaviours [3–5]. Alternatively, females may rely on endogenous stores to fuel their increased energetic load [6]. In contrast, DEE and exposure to predation risk for males often peaks during the mating season for species with female-only parental care [7]. Sex-related asymmetry in timing of energy allocation towards reproduction can also be augmented by sexual selection that drives the evolution of secondary sexual characteristics, including an energetically expensive increase in body and ornament size (sexual dimorphism), as well as sexual displays and contests between males for access to mates [8,9]. Given the trade-off between energy acquisition and risk of predation, many have hypothesized that sex differences in the timing of energy allocation towards reproduction would generate seasonal differences between sexes in time budgets with respect to foraging activity and risk-aversive behaviours.

Semi-fossorial animals face a direct trade-off between their ability to forage, which only occurs on the surface, and the risk of predation, which can be reduced by remaining below ground. Given this trade-off, energy demands of particular life-history stages may dictate the amount of time spent in the riskier above-ground environment. Time-spent above ground each day in golden hamsters (*Mesocricetus auratus*), for example, increases six- to eightfold coincident with the onset of lactation which suggests that energetic demand drives a concomitant increase in foraging effort and decrease in risk aversion [5]. Similarly, lactating Columbian ground squirrels (*Urocitellus columbianus*) spent more time above ground, more time foraging, and less time vigilant compared with chemically sterilized females that did not become pregnant [3]. In addition, males may engage in riskier behaviours (such as being above ground) if it affords them increased reproductive opportunities, even if energy demands are not substantially higher during the mating season [10].

Despite the widely held perception that above-ground activity in semi-fossorial species is shaped by the trade-off between predation risk and surface activities [3,11,12], studies examining this question are rare, partly because of inherent difficulties in observing individuals across time [13]. Because semi-fossorial mammals can mitigate the dangers of predation while above ground through vigilance and other risk-aversive behaviours, it is also not clear that time above ground will necessarily reflect a direct trade-off with the risk of predation. Additionally, while trade-offs between predation risk and foraging (or mating) opportunities can be important determinants of time spent above ground, they are certainly not the only factor. For example, weather conditions have long been known to alter activity levels of small mammals [14]. This may be due to increased costs of thermal exchange, the loss (or gain) of heat energy through convection, evaporation, conduction and radiation [15,16]. However, in at least some rodents, the effects of weather on activity are relatively slight and thought to relate more to predator avoidance than with physiological restrictions [17].

To date, most studies examining the potential trade-offs that influence behaviour in small mammals have relied on trapping data, and to a lesser extent, direct observations. Unfortunately, neither method permits the continuous tracking of individual behaviour throughout their annual life cycle. Here, we use the light loggers and accelerometers affixed to collars, to examine whether and how sex-specific reproductive requirements influence behaviour throughout the active season of a semi-fossorial hibernator, the arctic ground squirrel (*Urocitellus parryii*). Light collars provide us information on exposure to light and, in semi-fossorial animals, this can be used to assess when animals are above versus below ground [16]. Accelerometers measure movement along three orthogonal axis which can be used to calculate overall dynamic body acceleration (ODBA), an index of total movement that is highly correlated with activity-specific energy expenditure [18,19].

**Figure 1.**
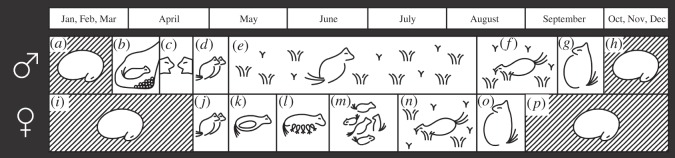
Male and female arctic ground squirrels differ in their timing of seasonally recurring life cycle events. Males (*a*) terminate heterothermy earlier than females, and (*b*) spend an average of 15–25 days below ground undergoing gonadal growth and spermatogenesis while they consume a food cache. Following emergence, (*c*) males establish territories and exhibit agonist interactions with other males as they compete to (*d*) mate with females. Following the mating season, (*e*) males undergo testicular regression and have several months during which they do not exhibit agonistic interactions before they (*f,g*) fatten in preparation for (*h*) resuming hibernation; a second interval of male–male aggression occurs in late summer/autumn. In contrast, females (*i*) hibernate longer but (*j*) mate within a few days of emergence; (*k*) gestation lasts approximately 25 days and (*l*) lactation lasts approximately 28 days. Females, but not males, (*m*) exhibit vigilance for predators while their newly emergent young are foraging but also (*n,o*) fatten rapidly prior to (*p*) initiating hibernation in mid- to late-August. Diagram based on data from [21–23].

As the northern-most hibernating small mammal, the arctic ground squirrel must reproduce, moult and fatten for the subsequent hibernation cycle during a comparatively short four to five month active season. In ground squirrels, the timing of peak energy allocation to reproduction differs between the sexes; the mating season for males lasts for only two to three weeks following emergence, whereas DEE of females increases during gestation and peaks during late lactation [20]. In addition to the energetic demands associated with gestation and lactation, female arctic ground squirrels undergo a moult and fatten rapidly following weaning, as they prepare for hibernation which commences in mid- to late-August; in contrast, males fatten and cache food in September and do not immerge into their hibernacula until early- to mid-October (figure 1 [21,22]). Based on these differences in timing, we predicted males would spend more time on the surface than females in April and early May as they defend territories and search for mating opportunities, whereas the high cost of gestation, lactation and pre-hibernation fattening would result in females being more active and spending more time above ground between mid-May and mid-August. Males, in contrast, might spend less time above ground when females are lactating and fattening as their own energy demands are low given they do not begin caching food or fattening until after females have initiated hibernation. Our predictions are based on the underlying assumption that when reproductive opportunities (for males) and energy demands (for both sexes) are low, individuals will decrease their risk of predation by spending more time below ground [3,6]. Alternatively, if being above ground serves functions beyond that of energy acquisition and seeking mating opportunities, then differences in activity levels between the sexes might be observed without a concomitant change in time above ground.

## 2. Methods

### 2.1. Study species and area

We investigated above-ground activity in two nearby populations of arctic ground squirrels living above 68° N, north of the Brooks Range, Alaska. The first site, located near Atigun River (hereafter: Atigun; 68°27′ N, 149°21′ W; elevation 812 m), lies approximately 20 km south of the second site, located adjacent to Toolik Lake (hereafter: Toolik; 68°38′ N, 149°38′ W; elevation 719 m). Population density is higher at Atigun, owing, in part, to the sandy substrate that is well-drained and suited to burrowing; suitable substrate for burrowing is more dispersed at Toolik. Earlier loss of snow cover at Atigun typically results in earlier (9–13 days) timing of spring emergence from hibernation and reproduction, relative to Toolik [22,24]. Risk of predation for adult ground squirrels at our study sites is higher on the surface where predators include red fox (*Vulpes vulpes*), grey wolf (*Canis lupus*), golden eagles (*Aquila chrysaetos*), northern harrier (*Circus cyaneus*) and short-eared owls (*Asio flammeus*); common ravens (*Corvus corax*) will predate juveniles but no attempted predation of adults has been observed. Ermine (*Mustela erminea*) will attack ground squirrels on the surface but may also consume juveniles, and possibly adults, in their burrows. Other ecological site characteristics are described in [22].

Following the termination of heterothermy in spring, male arctic ground squirrels remain below ground for a three to five week interval during which they consume a food cache to regain body mass lost during hibernation and undergo testicular growth and maturation (figure 1 [21,25]). Males emerge in mid-April in anticipation of female emergence, which typically occurs 11–14 days later [21,22,26]. Male–male aggression, physical confrontations and wounding are common during the mating season [27]. Males intercept and mate-guard newly emergent females that become pregnant within a few days of emergence; gestation lasts for approximately 25 days, and lactation is another approximately 28–35 days [23,28].

Unlike males, females do not cache food and, with the exception of early gestation when they continue to lose body fat, they appear to fuel their reproduction using energy gained concurrently through foraging [21]. Although DEE has not been measured in arctic ground squirrels, DEE of females in other ground squirrel species peaks during late lactation and exceeds DEE of males at any time during the active season [1]. In addition, females are delivering energy to pups as milk during lactation, but this energy is not included in the DEE measurement. Once their young have been weaned, females undergo a moult and fatten; autumn immergence occurs in August (figure 1 [21,22]). Fattening in arctic ground squirrels is not associated with a decrease in lean mass-specific resting metabolic rate (RMR) [29] which suggests their foraging effort is likely to be higher at this time of year relative to males that fatten and cache food later in the autumn and immerge in early- to mid-October [21,22].

### 2.2. Light loggers

We used the light loggers to determine whether individuals were above ground (light) versus below ground (dark) from first emergence in the spring of 2014 until immediately prior to when females begin to immerge in autumn. In the second year of our study, we have data from first emergence until early August; however, we do not include data beyond 9 June in our models (see below) owing to low sample sizes. Two types of loggers were deployed; BAS model MK7290 light loggers (Biotrack Ltd, Dorset, UK), which record light levels every 2 min, and Intigeo-C56 light loggers (1 g; Migrate Technology Ltd, Cambridge, UK), which record light every minute and then save the highest measured value per 5 min interval. For further details on methods using light loggers, see [16]. We measured above-ground versus below-ground activity patterns in 23 females (13 Atigun; 10 Toolik) and 15 males (nine Atigun; six Toolik) in 2014 and 24 females (19 Atigun; five Toolik) and 17 males (10 Atigun; seven Toolik) in 2015. Not all animals were tracked throughout the entire interval, as we continuously deployed and downloaded collars; on average, we obtained 52 ± 25 (s.d.) days of data per individual during the active season in 2014 and 39 ± 12 days in 2015.

Data on timing of emergence were obtained from 16 females (10 Atigun and six Toolik) and nine males (five Atigun and four Toolik) in 2014; these individuals were equipped with either collars or implanted body temperature (*T*_b_) loggers (for details on assessing timing using *T*_b_ loggers, see [23]). In 2015, we obtained emergence data for 14 females (nine Atigun and five Toolik) and nine males (four Atigun and five Toolik). We captured animals every four to six weeks throughout the active season to download light collars. Following capture, animals were anaesthetized by a 3–5 min exposure to isoflurane vapours, identified using unique ear and passive-integrated transponder tags, weighed and assessed for sex and reproductive status. Blood samples were also collected to investigate the relationship between activity and thyroid hormone level, which we report elsewhere [30]. In 2014, we measured body mass at four different stages of the breeding season including emergence (20 April–3 May), early- to mid-lactation (2 June–16 June), post-lactation (1 July–7 July) and late in female fattening (8 August–14 August). In 2015, we obtained measurements at the same time of year during emergence and mid-lactation, but not during the post-lactation and female fattening intervals. To guard against effects of handling/anaesthesia on behaviour, we excluded above/below-ground data from individuals on the days when their collars were deployed or downloaded.

### 2.3. Accelerometers

In 2014, we found little difference between the sexes in time spent above ground each day across the active season, with the exception of parturition/early lactation when females spent less time above ground (see Results). This lack of sex differences in time above ground might indicate that differences in reproductive requirements do not influence daily above-ground activity or it may indicate that time above ground is not reflective of movement or foraging activity on the surface. To differentiate between these two possibilities, we deployed collar-mounted accelerometers (less than 3 g, axy-3 loggers, TechnoSmart Europe srl., Rome, Italy) on squirrels at the Atigun site in 2015; we successfully recaptured and obtained data from six males and six females, all of which were also equipped with light loggers (collar with epoxy-mounted light logger and shrink-wrapped accelerometer: approx. 8 g, less than 5% of body mass). Accelerometers were deployed from 29 April (early gestation) to 10 June (late lactation) and were programmed to record in the *X*-, *Y*- and *Z*-axis once per second. For each axis, the static effect of gravity on acceleration was removed from the acceleration data by subtracting the 11 s running mean. We then calculated ODBA using the method of [18] that involves summing of the absolute values of the calculated dynamic acceleration for each axis. Although ODBA is typically calculated using measurements of acceleration at a frequency of 10 Hz or higher, sampling frequencies as low as 1 Hz provide reasonable estimates of energy expenditure, even in small animals [31].

### 2.4. Environmental data

We measured a variety of environmental parameters known to influence operative temperature, which includes the effects of convective and radiant heat transfer, at both of our study sites using weather stations. At Atigun, we collected environmental data (incident solar radiation, ambient temperature, wind speed and rainfall), using a Hobo U30-NRC weather station (Onset Computer Corporation, Bourne, MA). For Toolik, we acquired the data on these same parameters from the Toolik Field Station Environmental Data Center (http://toolik.alaska.edu/edc/index.php) weather station. Precipitation data were collected using tipping buckets at both sites that do not record precipitation that falls as snow. However, we included a categorical variable (yes/no) to account for major snowfall events that occurred during the daylight hours; observers were in place to record such events throughout the study. For our statistical models, we calculated the average values for each environmental parameter (or sum for precipitation) between 08.00 and 21.00 each day, the timeframe when squirrels are active on the surface [16]; use of daily averages produced essentially the same results.

### 2.5. Statistical analyses

Statistical analyses were performed using SAS v. 9.4 (SAS Institute, Cary, NC); for all models, we examined normality and assessed goodness of fit, using the QQ plots. We compared body mass at different stages of the active season separately within each sex, using linear mixed models that included individual (animal identification) as a random effect. Having found a significant effect of stage, we subsequently made all pairwise comparisons using *post hoc* Tukey–Kramer tests. We also used mixed models to investigate whether there were differences between years within the same sex at each time of year (life-history stage); results for these analyses were the same, regardless of whether a Bonferroni correction for multiple comparisons was applied. We compared environmental conditions between sites using a paired (by day) non-parametric test, the Wilcoxon signed-rank test.

For each year and site, we examined the effects of sex and environmental conditions on time spent above ground each day and mean daily ODBA using mixed models with ID included as a random effect. To account for nonlinearity across the breeding season, we applied penalized B-splines (hereafter: p-splines) using mixed model methodology and allowed the splines to vary by group (sex). Environmental parameters used in the models included average daily wind speed (m s^−1^), incident solar radiation (J cm^−2^ h^−1^) and ambient temperature (°C); we also included an interaction between wind speed and ambient temperature (i.e. a wind chill effect). Rainfall was included as a categorical variable (either 0 mm, 0–2 mm, or more than 2 mm rain per day), as were snowfall events (yes/no). For 2014, we included data from 3 May–31 July in our models, which includes gestation, lactation, moult and fattening of reproductive females; we truncated the season to include only the timeframe when no animals were hibernating and when sample sizes were sufficient for parameter estimation. For our Atigun River site, we also analysed the data between 21 April and 29 April (2014) separately, using a linear mixed model (no spline), excluding individuals that had not yet terminated hibernation; this timeframe includes the part of the season when males establish territories, seek out females for mating and mate-guard; sample sizes for non-hibernating Toolik animals during this interval were too small for a separate analysis. In 2015, we model only data for Atigun because sample sizes for Toolik were small across the entire active season; qualitative results from Toolik were similar to those at Atigun despite the small sample size (*n* = 3 and 4 for females and males, respectively). For 2015, our models for time spent above ground each day (light loggers) and mean daily ODBA (accelerometers) included data between 27 April and 9 June; sample sizes later in the season were too low.

## 3. Results

### 3.1. Body mass

Body mass varied among life-history stages in females for both years of study (2014: *F*_3,58_ = 77.4, *p* < 0.0001; 2015: *F*_1,15_ = 49.98, *p* < 0.0001) such that females gained weight across the season (table 1). In the first year of study (2014), female body mass was not affected by site (*F*_1,58_ = 1.56, *p* = 0.21) or by the interaction between site and stage (*F*_3,58_ = 0.60, *p* = 0.62). In the second year of study, however, female body mass was significantly affected by the interaction between site and stage (site: *F*_1,15_ = 0.80, *p* = 0.39; site × stage: *F*_1,15_ = 6.53, *p* = 0.02) such that body mass of females at Toolik was greater than females at Atigun during the mating season in April (Atigun: 489 ± 92 (s.d.); Toolik: 565 ± 78), but not during early lactation in June (Atigun: 611 ± 72; Toolik 631 ± 20). Lactating females (June) were significantly heavier in 2015 relative to 2014 (*F*_1,43_ = 9.86, *p* = 0.003; table 1).

**Table 1.** Mean body mass (±s.d.) of female and male arctic ground squirrels at sites located adjacent to Atigun River and Toolik Lake in 2014 and 2015; sample sizes are shown in brackets. Different letters indicate significant differences between months within sex-year groupings. Female body mass data for July and August 2015 were not included in statistical analyses owing to low sample size. Asterisks indicate differences between years within sex-month groupings.

	females		males	
	2014	2015	2014	2015
April	516 ± 67 g (32)^a^	507 ± 93 g (31)^a^	677 ± 123 g (30)^a^	681 ± 144 g (23)^a^
May	—	564 ± 81 g (22)^ab^	—	—
June	565 ± 64 g (31)^b^	625 ± 72 g (21)^b,*^	685 ± 80 g (12)^a^	712 ± 79 g (13)^a^
July	623 ± 79 g (23)^c^	803 ± 19 g (3)	774 ± 78 g (16)^b^	763 ± 76 g (17)^ab^
August	791 ± 68 g (14)^d^	794 ± 157 g (5)	805 ± 97 g (12)^b^	839 ± 81 g (9)^b^

Body mass of males changed with life-history stage in both 2014 and 2015 (2014: *F*_3,30_ = 19.17, *p* < 0.001; 2015: *F*_3,27_ = 5.54, *p* = 0.004), but was not affected by site (2014: *F*_1,30_ = 0.67, *p* = 0.42; 2015: *F*_1,27_ = 1.44, *p* = 0.24) or by the interaction between site and stage (2014: *F*_1,30_ = 0.61, *p* = 0.62; 2015: *F*_3,27_ = 1.03, *p* = 0.39) in either year. Body mass of males increased across the active season (table 1) but did not differ significantly between years for any life-history stage. In both years, body mass of males was similar to morphometrically smaller females in early August, indicating they had not yet begun to fatten for hibernation (table 1). Mass of juveniles in August differed between years (*F*_43,1_ = 8.11; *p* = 0.007) but not between the sexes (*F*_1,43_ = 2.01; *p* = 0.16); juveniles were 51 g lighter in 2014, relative to 2015, despite being weighed 7 days later in the year, on average.

### 3.2. Spring phenology

In the first year of the study, mean date of spring emergence for Atigun males was 16 April (range: 13–21 April), slightly earlier, but overlapping with Atigun females (mean: 20 April; range: 13–26 April). Similarly, mean spring emergence for males at Toolik was on 18 April (range: 4–26 April), whereas mean emergence for females was 26 April (range: 24–29 April). In the second year of the study, males and females from Atigun emerged from their hibernacula on 9 April (range: 8–15 April; *n* = 5) and 18 April (range: 13–24 April; *n* = 9), respectively. At Toolik, males and females emerged slightly later, on 16 April (range: 12–20 April; *n* = 2) and 23 April (range: 19–24 April; *n* = 5), respectively.

### 3.3. Time above ground in 2014

**Figure 2.**
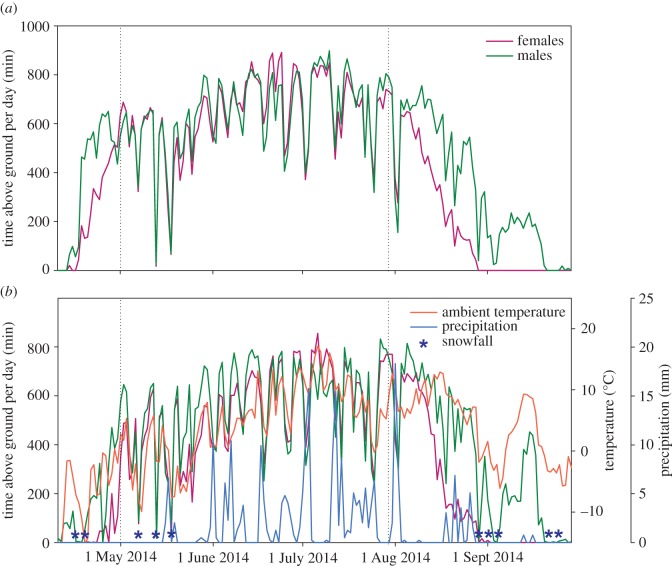
Mean duration (minutes) spent above ground each day in 2014 for female (purple) and male (green) arctic ground squirrels at our field sites at (*a*) Atigun River and (*b*) Toolik Lake. Time above ground varies substantially from one day to the next owing to the effects of environmental conditions known to affect thermal exchange including mean ambient temperature (red line), total daily precipitation (blue line), blizzard events (blue asterisks), solar radiation (not shown) and wind speed (not shown). The vertical dashed lines indicate the start and end dates for the data used in the mixed model, which excludes intervals when any individuals were hibernating.

At both sites, time spent above ground each day varied widely from one day to the next (figure 2). Although males appear to be above ground more than females in late April, the mean above-ground activity of females during this interval is influenced by females that have not yet terminated hibernation and emerged to the surface. When hibernating individuals are removed from the dataset, there is no significant difference between males and females in their durations spent above ground from 21 to 30 April at Atigun (*F*_78,1_ = 3.01, *p* = 0.09).

Between 3 May and 31 July 2014, females and males at Atigun spent 634 ± 165 min (s.d.) (CV = 0.26) and 640 ± 170 min (CV = 0.27) above ground each day, respectively. During this same timeframe, animals at Toolik spent substantially less time above ground; females were above ground 534 ± 183 min (CV = 0.34) each day, whereas males were above ground for 559 ± 193 min (CV = 0.34). Toolik was significantly cooler (−1.3 ± 0.1°C (s.e.), *S* = −3391.5, *p* < 0.0001) and received more precipitation (0.37 ± 0.16 mm day^−1^, *S* = 280.5, *p* = 0.01) compared with Atigun, but the two sites did not differ in terms of wind speed (*p* = 0.23) or solar radiation (*p* = 0.42).

**Figure 3.**
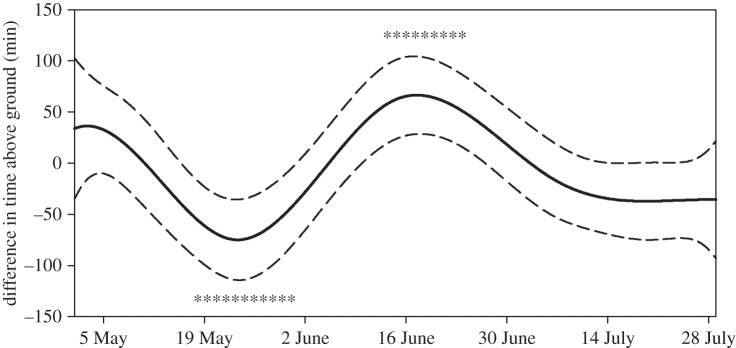
Predicted differences in p-splines between the sexes for time spent above ground each day by arctic ground squirrels across the 2014 active season at Atigun River. The solid line shows the mean prediction and the dotted lines, the 95% CIs. Negative values indicate females spend less time above ground compared with males. Asterisks indicate time intervals with significant sex differences (*p* < 0.05; Holm's step-down adjustment for multiple comparisons).

Our model for Atigun animals that encompassed the timeframe when both sexes were active indicated that environmental conditions significantly affected time spent above ground (table 2); similar results were obtained for the smaller dataset from Toolik (electronic supplementary material, table S1). Parameter estimates for environmental variables were consistent with the prediction that squirrels reduce their time spent above ground when operative temperatures decrease; above-ground activity increased with increasing temperature, increasing solar radiation and decreasing wind speed, and was negatively affected by precipitation (table 2 and electronic supplementary material, S1). Our models also predicted that time spent above ground each day differed between the sexes, with females spending less time above ground during parturition and early lactation, but more time above ground during late lactation (Atigun p-spline: *p* = 0.0001, sex: *p* = 0.49, sex × p-spline: *p* < 0.0001; Toolik p-spline: *p* = 0.001, sex: *p* = 0.0001, sex × p-spline: *p* < 0.0001; figure 3 and electronic supplementary material, S1). The timeframe during which females at Toolik decreased their activity was later than at Atigun, consistent with the differences in spring emergence phenology between sites. However, unlike Atigun, females from Toolik spent less time above ground than males shortly after hibernation had terminated (1–5 May; electronic supplementary material, figure S1).

**Table 2.** Parameter estimates for environmental variables, 95% CIs and *p*-values from a mixed model examining the factors influencing time arctic ground squirrels spent above ground each day (min) at Atigun River between 3 May and 29 July 2014. The mixed model also included p-splines that varied by sex allowing for nonlinear changes across the time interval. A total of 1143 days of data from 24 individuals were included in the model.

parameter		estimate [95% CI]	*p*-value
temperature (°C)		7.8 [4.8, 10.7]	<0.0001
wind speed (km h^−1^)		−10.7 [−18.9, −2.5]	0.01
temp × wind speed		1.1 [0.27, 1.37]	0.009
solar radiation (lux)		0.45 [0.39, 0.51]	<0.0001
rain (mm day^−1^)	>2 mm	−114.1 [−135.3, −92.9]	<0.0001
	0–2 mm	−40.8 [−54.8, −26.8]	<0.0001
	0	0	
snowfall	no	269.3 [234.7, 303.9]	<0.0001
	yes	0	

### 3.4. Time above ground and overall dynamic body acceleration in 2015

**Figure 4.**
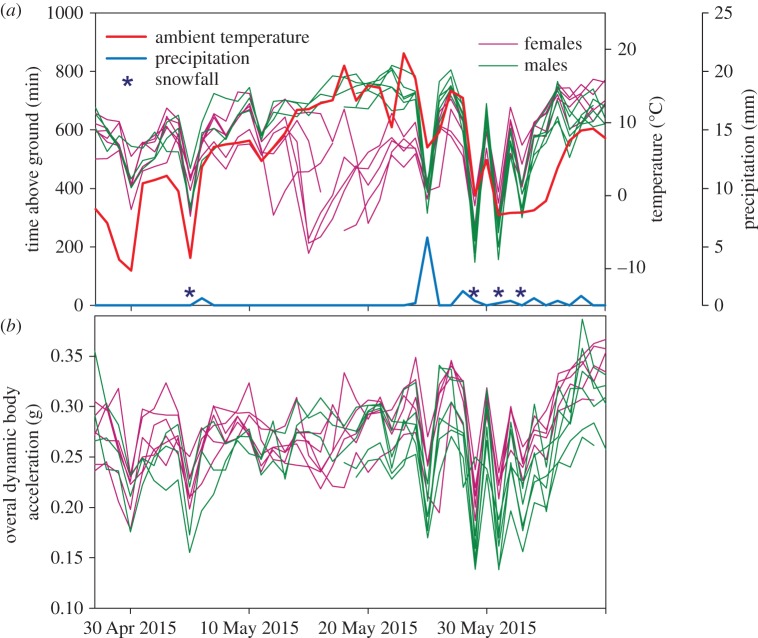
(*a*) Minutes spent above ground each day and (*b*) mean daily ODBA in 2015 for female (purple lines) and male (green lines) AGS at our field site at Atigun River. Individuals for which there is no corresponding acceleration data are not shown. Time above ground and ODBA were significantly affected by environmental conditions known to affect thermal exchange conditions including, total daily precipitation (blue line), snowfall events (blue asterisk), ambient temperature (red line; ODBA only) solar radiation (not shown; time only) and wind speed (not shown).

**Figure 5.**
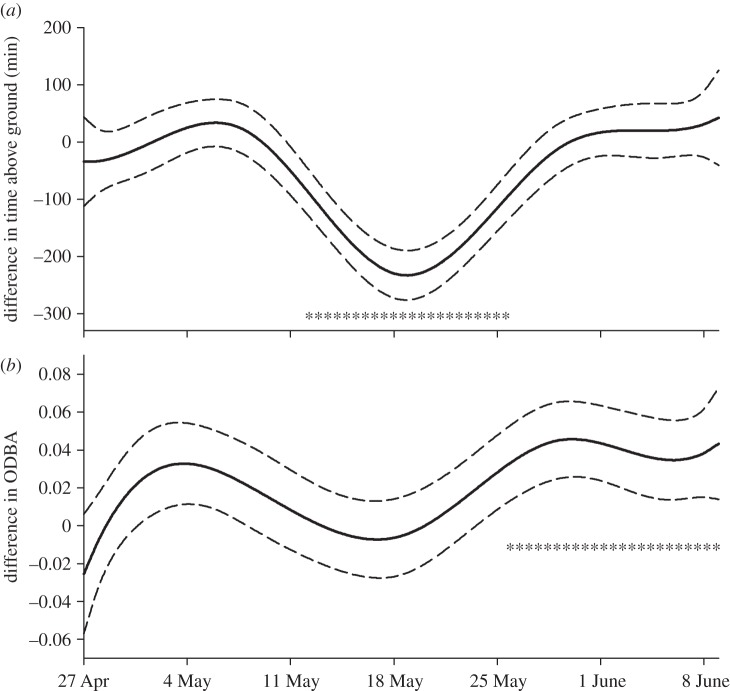
Predicted differences between the sexes in p-splines for (*a*) time (min) spent above ground each day and (*b*) mean daily ODBA of arctic ground squirrels from Atigun River, 27 April–9 June 2015. The solid lines show the mean predictions and the dotted lines, the 95% CIs. Negative values indicate females spend less time above ground or have lower ODBA compared with males. Asterisks indicate time intervals with significant differences (*p* < 0.05; Holm's step-down adjustment for multiple comparisons).

Similar to 2014, day-to-day variation in time above ground at Atigun in 2015 was high, with reduced activity on wet and/or snowy days (figure 4*a* and table 3). However, 2015 was a drier year with only 120 mm of rain falling between 1 May and 1 August compared with 209 mm in 2014. Similar to 2014, our 2015 model for time spent above ground each day also predicted females spent less time on the surface compared with males during parturition and early lactation at Atigun (p-spline: *p* < 0.0001, sex: *p* = 0.67, spline × sex: *p* < 0.0001). The size of this effect, however, was fourfold higher in 2015 (figure 5*a*). Similar to time spent above ground, model results indicate ODBA is affected by thermal exchange conditions (table 4). However, sex differences in p-splines for ODBA across the season did not parallel differences seen in activity; female ODBA was not different from that of males during early lactation, but female ODBA was higher between 26 May and 9 June, which corresponds with late lactation (figures 4*b* and 5*b*).

**Table 3.** Parameter estimates for environmental variables, 95% CIs and *p*-values from a mixed model examining the factors influencing time arctic ground squirrels spent above ground each day (min) at Atigun between 27 April and 9 June 2015. The mixed model also included p-splines that varied by sex allowing for nonlinear changes across the time interval. A total of 686 days of data from 20 individuals were included in the model.

parameter		estimate [95% CI]	*p*-value
temperature (°C)		3.3 [−0.8, 7.4]	0.12
wind speed (km h^−1^)		−16.3 [−26.5, −6.0]	0.002
temp × wind speed		1.1 [0.0, 2.2]	0.09
solar radiation (lux)		0.30 [0.19, 0.41]	<0.0001
rain (mm day^−1^)	>2 mm	−140.6 [−204.5, −76.7]	<0.0001
	0–2 mm	−10.3 [−20.9, 10.4]	0.33
	0	0	
snowfall	no	160.2 [131.4, 193.0]	<0.0001
	yes	0

**Table 4.** Parameter estimates for environmental variables, 95% CIs and *p*-values from a mixed model examining the factors influencing average daily ODBA of arctic ground squirrels at Atigun between 27 April and 9 June 2015. The mixed model also included p-splines that varied by sex allowing for nonlinear changes across the time interval. A total of 400 days of data from 12 individuals were included in the model.

parameter		estimate [95% CI]	*p*-value
temperature (°C)		0.0020 [0.0006, 0.0034]	0.007
wind speed (km h^−1^)		−0.0069 [−0.011, −0.0037]	<0.0001
temp × wind speed		−0.0004 [−0.0008, −0.000]	0.0048
solar radiation (lux)		0.00003 [−0.00001, 0.00007]	0.15
rain (mm day^−1^)	>2 mm	−0.0485 [−0.0697, −0.02728]	<0.0001
	0–2 mm	−0.0029 [−0.0096, 0.0058]	0.41
	0	0	
snowfall	no	0.0392 [0.0284, 0.0500]	<0.0001
	yes	0	

**Figure 6.**
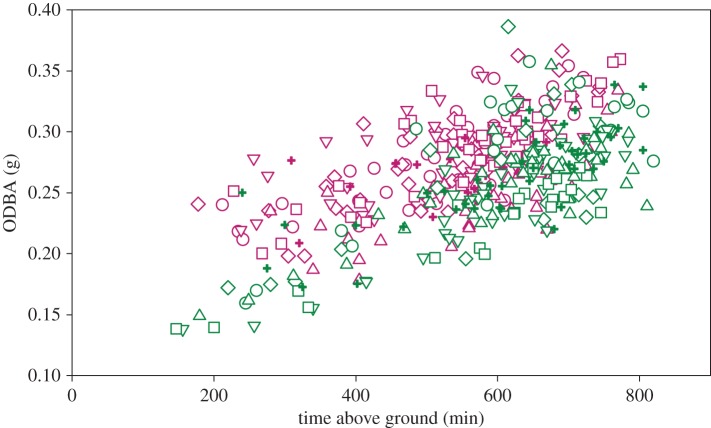
Mean daily ODBA versus time spent above ground each day in female (purple) and male (green) AGS in 2015. Different symbols indicate different individuals.

**Figure 7.**
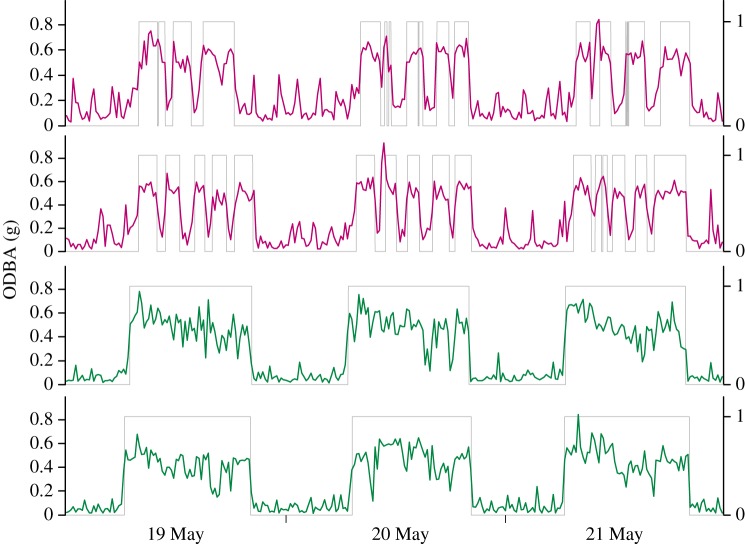
Time spent above/below ground (grey line; 1, above ground; 0, below ground) and ODBA (averaged in 10 min blocks; colour line) on 19–21 May 2015 in two representative females (top panels) and two representative males (bottom panels). Bouts spent below ground (darkness) that were less than 5 min are not shown.

The difference in patterns occurs because females have higher mean daily ODBA for a given amount of time spent above ground compared with males (figure 6). Examination of within-day variation in time spent above ground and ODBA revealed that, during early lactation, females make frequent forays below ground throughout the day, presumably to warm and nurse young (figure 7). Time spent above ground by females during early lactation of the prior year (2014) was also interrupted by short intervals spent below ground (not shown); however, these intervals were much shorter compared with 2015 resulting in a smaller difference between the sexes in terms of time spent above ground per day. Examination of ODBA indicates lactating females also exhibited sporadic bouts of below-ground activity between the hours of approximately 22.00 and approximately 08.00, when they were not on the surface (figure 7).

## 4. Discussion

Life-history theory predicts that sex-based differences in the timing of reproduction and in when energy is allocated towards reproduction should be manifest in sex-specific differences in activity patterns. We predicted that trade-offs between above-ground foraging activity and risk of predation would lead to intraspecific differences within arctic ground squirrels in daily surface activity across the active season with females spending more time above ground than males during lactation, which is energetically expensive. Although we found some evidence for this in late lactation, females tended to spend less time, not more, above ground between parturition and early lactation, presumably so they could provide maternal care to their offspring which are born hairless and incapable of independent thermoregulation; soil surrounding burrows in the Arctic remain frozen until late summer [32].

Our results contrast with previous studies of semi-fossorial mammals which suggested that high energy demands during lactation will drive a concomitant increase in time spent above ground by females [3,6]. Instead, we found that thermal exchange conditions played the most important roles in determining time spent above ground each day, and the energy demands of lactation were offset to a large degree by greater movement (ODBA) of females while on the surface, which we assume is indicative of greater foraging effort. Although females spent less time on the surface than males during early lactation in both years, the size of this effect was fourfold higher in 2015 when conditions were drier (less rain and fewer snowfall events). Females were 12% heavier at this stage of the breeding season in 2015, relative to 2014, indicating they probably had greater lipid and/or protein stores, and we suggest they used this stored capital to increase time spent below ground, nursing and warming their newborn offspring.

### 4.1. Thermoregulatory costs

Predation has long been considered a major selective force in the evolution of behaviour and behavioural plasticity [33], yet our study demonstrates that it is not appropriate to assume behaviour necessarily reflects a simple trade-off between risk of predation, as estimated by time spent above ground, and energy acquisition. Thermoregulatory conditions are clearly an important driver of activity patterns throughout the active season in arctic ground squirrels, with more than 50% of the variation in time spent above ground and ODBA explained by day-to-day differences in weather variables. This finding is surprising in the light of a recent study on red squirrels that indicated the effects of reproductive stage can overwhelm environmental effects on DEE, as measured using the doubly labelled water approach [2]. Studies of other ground squirrels also indicate that reproductive stage is an important driver of energy expenditure with DEE peaking during late lactation [1]. This is consistent with the higher levels of ODBA we measured for females in late lactation, given that activity-specific energy expenditure makes up a substantial portion of DEE [31]. However, it is important to note that this pattern (higher energy expenditure during late lactation) could become obscured by effects associated with daily variation in weather conditions if DEE is measured over short, 2–4 day intervals, as is typically the case for the doubly labelled water approach. The importance of environmental conditions in our study may be owing, in part, to the high variability in weather-related differences in thermal exchange that characterize the arctic summer; more work is needed to better document the impacts of weather conditions on temperate and tropical species.

Our findings are consistent with recent studies indicating thermoregulatory costs can play a much greater role in driving the timing and duration of daily behaviours than previously appreciated. For example, nocturnal mice will become diurnal when challenged by cold, because activity during the warmer daytime combined with a period of rest in a buffered environment during the colder night reduces energy expenditure [34]. Similarly, we have hypothesized that it is this thermoregulatory advantage of ‘daytime’ activity that leads to persistent, entrained daily rhythms of physiology and behaviour during the polar day in arctic ground squirrels [15,35].

For much of our study, we found that both sexes spent similar amounts of time above ground, but females were consistently more active, as indicated by higher levels of ODBA. The difference between the sexes in ODBA may indicate that males are using risk-aversive behaviours to a greater degree than females during the interval in which their energetic demand is low. There is widespread evidence in ungulates that males will have higher vigilance at the expense of reduced foraging effort relative to females during lactation [36,37]. Small mammals are also known to engage in behaviours that mitigate the risk of predation while foraging. For example, Thorson *et al.* [38] found that thirteen-lined ground squirrels (*Ictidomys tridecemlineatus*) will abandon foraging effort at much higher food densities when the patch is located further away from escape burrows. Similarly, van der Merwe & Brown [39] showed that use of food patches by Cape ground squirrels (*Xerus inauris*) was governed primarily by proximity to burrows and open sight lines. In one of the few studies to investigate seasonal sex-related changes in the time budgets of a small mammal across the season, however, Ebensperger & Hurtado [40] found no evidence for seasonal changes or sex differences in time spent vigilant or foraging in degus (*Octodon degus*). We suggest that fine-grained data from increased use of biologging technology, combined with experimental manipulations at the level of the individual, will help shed light on the proximate and ultimate drivers of behaviour and energy expenditure in free-living small mammals [41].

This study indicates that male ground squirrels do not reduce their risk of predation outside of the mating season by spending more time below ground. However, it is not clear what these animals are doing while above ground, particularly given their ODBA is lower than females during gestation and late lactation. It is possible that time above ground serves some sort of social function, such as the establishment and/or persistence of territories [42]. However, arctic ground squirrels exhibit territorial behaviour and male/male aggression only in the early spring and late autumn [27], which suggests this is likely to be unimportant during the interval when females are lactating and fattening. The additional time spent above ground may be simply to loaf/bask in the sun. In small mammals, basking behaviour has been commonly reported as a means of passive rewarming from torpor [43], but it is not often considered as a thermoregulatory mechanism despite the high thermoneutral zone of many small mammals. Basking has, however, previously been reported in antelope ground squirrels [44] (*Ammospermophilus leucurus*) and yellow-bellied marmots [45] (*Marmota flaviventris*). Examination of ODBA data, however, suggests that males are continually active throughout the day while above ground (figure 5). Even if males are not basking they may still benefit from being on the surface because, at our study site, the active soil layer typically does not thaw until late July and therefore burrow temperatures remain at or below freezing for most of the summer months [32]. Thus, while a below-ground nest may provide a thermal refuge when it is cold or raining on the surface, it may be advantageous to escape the cool burrow and remain above ground when thermoneutral conditions prevail on the surface.

Our results suggest day-to-day variability in time above ground and activity levels in arctic ground squirrels, in large part, reflects strategies related to behavioural thermoregulation rather than being associated with attempts to mitigate the risk of predation. However, we did find rather substantial (approx. 1 h) differences between our study sites in the amount of time spent above ground each day in 2014, and site differences in thermoregulatory conditions explained only approximately 25% of this difference. This suggests that time above ground is also likely to be affected by other factors such as population density, forage availability/quality or risk of predation. Establishing the relative importance of these other factors is likely to require field experiments designed to manipulate these parameters.

### 4.2. Annual differences

Although males and females differed in their timing of emergence from hibernation, we found relatively small sex-based differences in time spent above ground each day across life-history stages in the first year of our study. This finding was not consistent across years, however, as females spent substantially less time above ground during early lactation in the second year of our study. The drop in activity of individual females did not all occur on the same day and was not linked to changes in weather events, suggesting that it may have been driven by the need to give birth, nurse and provide thermoregulation for young during the early stages of lactation; ground squirrels are born hairless and the onset of endothermy is a gradual process during development [46]. Within-day patterns of above-ground activity during this interval were consistent with this hypothesis; females had regular intervals of above-ground activity during which movement was high, interrupted by below-ground episodes with little movement. These below-ground episodes also occurred in the first year of our study (2014; not shown), but were much shorter, such that there was a fourfold smaller difference between males and females in total time spent above ground each day. Although the cause of the between-year differences in above ground activity is unknown, females were in better body condition in early lactation during the second year of our study and we propose this may have allowed them to allocate more time to maternal behaviours, including warming and nursing their young. Juveniles born in 2015 had higher body mass in early August compared to the 2014 cohort, which may have been owing to interannual differences in maternal care. However, we cannot rule out the possibility that this difference may have been associated with better post-weaning foraging conditions or other disparities between years.

## 5. Conclusion

In hibernators, differences between the sexes in how and when energy is allocated towards reproduction have led to substantial sex-related variation in the seasonal onset and termination of heterothermy [47]. We anticipated these sex differences in reproductive requirements would also influence time spent above ground each day across the active season. Interestingly, the observed effects were context-dependent; females reduced their time above ground during early lactation, but this effect was much larger in the second year, possibly because they were in better body condition. We also found that although activity levels were correlated with time spent above ground, the relationship differed between the sexes with females being more active per unit time above ground. Thus, in addition to the energetic costs of producing milk during late lactation, females also have higher activity-specific energy expenditure than males during this interval. The sex-dependent plasticity we observed suggests males and females may react differently to changing environmental conditions with females less able to absorb changes that result in a further increase in foraging effort. Finally, we found that time spent above ground and ODBA (activity-specific energy expenditure) varied substantially from one day to the next for both sexes and most of this variation was attributable to weather-driven changes in thermal exchange conditions. Our results highlight the need to consider elements beyond predator avoidance and energy acquisition in studies focused on understanding how animals use space and time.

## Supplementary Material

Table S1. Parameter estimates for environmental variables Figure S1. Predicted differences between the sexes in p-splines

## Data accessibility

Data available from the Dryad Digital Repository (doi:10.5061/dryad.2dv6n). Weather data for Toolik Lake available from Toolik Field Station Environmental Data Center: http://toolik.alaska.edu/edc/abiotic_monitoring/index.php
