# Supplementary material for: The secret life of ground squirrels: accelerometry reveals sex-dependent plasticity in above-ground activity
Source: R Soc Open Sci. 2016 Sep 28;3(9):160404. doi: 10.1098/rsos.160404 (PMC5043325; doi:10.1098/rsos.160404)
Supplement: Table S1. Parameter estimates for environmental variables Figure S1. Predicted differences between the sexes in p-splines [file rsos160404supp1.pdf]

Table S1. Parameter estimates for environmental variables, 95% confidence intervals, and P-values from a mixed model examining the factors influencing time arctic ground squirrels spent above ground each day (min) at Toolik Lake between 3 May and 29 July, 2014. The mixed model also included p-splines that varied by sex allowing for non-linear changes across the time interval. A total of 993 days of data from 18 individuals were included in the model.

Parameter		**Estimate [95% CI]**	**P-Value**
Temperature (°C)		14.3 [9.8, 18.8]	<0.0001
Wind Speed (km/h)		-28.7 [-40.9, -16.5]	<0.0001
Temp*Wind Speed		1.2 [-0.2, 2.4]	0.09
Solar Radiation (lux)		0.57 [0.49, 0.64]	<0.0001
Rain (mm/day)	>2mm	-71.6 [-97.0, -46.4]	<0.0001
	0-2mm	20.0 [-1.7, 42.7]	0.07
	0	0	
Snowfall	No	149.6 [87.7, 211.5]	<0.0001
	Yes	0	

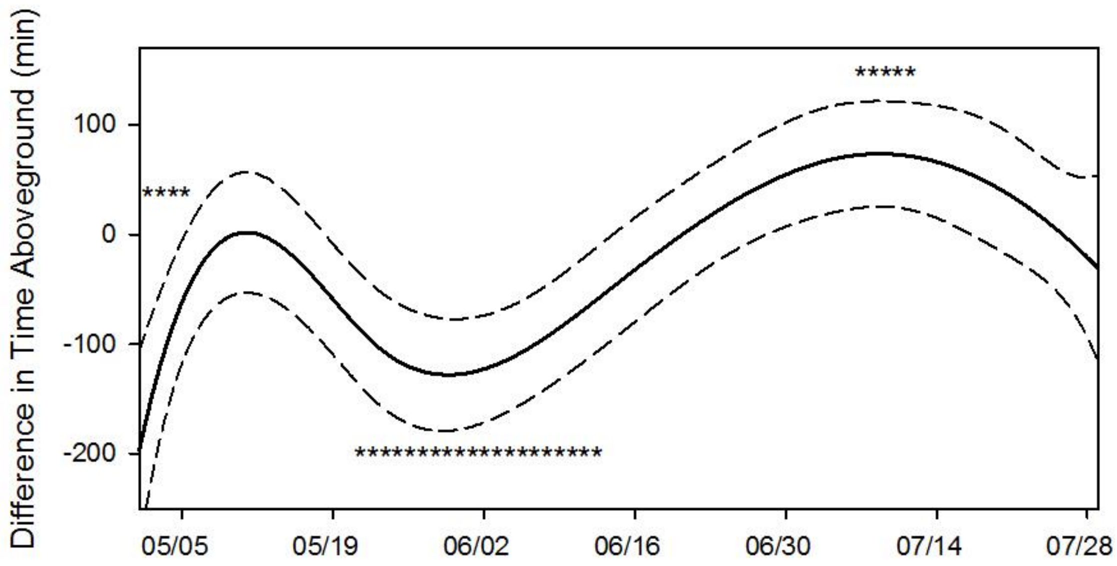

Figure S1. Predicted differences between the sexes in p-splines for time spent above ground each day by arctic ground squirrels across the 2014 active season at Toolik Lake. The solid line shows the mean prediction and the dotted lines, the 95% confidence intervals. Negative values indicate females spend less time above ground compared to males. Asterisks indicate time intervals with significant sex-differences ($P < 0.05$; Holm's step-down adjustment for multiple comparisons).
